# Clinical evaluation of photodynamic therapy for oral leukoplakia: a retrospective study of 50 patients

**DOI:** 10.1186/s12903-023-03791-5

**Published:** 2024-01-03

**Authors:** Yanting Wang, Haonan Tang, Keyi Wang, Yuping Zhao, Juanyong Xu, Yuan Fan

**Affiliations:** 1 Department of Oral Mucosal Diseases, The Affiliated Stomatological Hospital of Nanjing Medical University, Nanjing, China; 2 Jiangsu Province Key Laboratory of Oral Diseases, Nanjing Medical University, Nanjing, China; 3 Jiangsu Province Engineering Research Center of Stomatological Translational Medicine, Nanjing, China

**Keywords:** Photodynamic therapy, 5-aminolevulinic acid, Oral leukoplakia, DNA ploidy analysis, Side effects, Recurrence

## Abstract

**Background:**

Topical photodynamic therapy (PDT) has demonstrated encouraging results in the treatment of oral leukoplakia (OLK). However, data on the clinical efficacy of PDT in Chinese patients with OLK are still limited.

**Methods:**

Fifty patients diagnosed with OLK were enrolled, including patients with various dysplastic tissues. All patients received topical PDT with 5-aminolevulinic acid (5-ALA) as a photosensitizer. Clinical efficacy was evaluated 4 weeks after treatment. Follow-up was performed every 3 months during the first year and every 6 months during the second year.

**Results:**

The overall response rate was 68% (34/50): 12% (n = 6) complete and 56% (n = 28) partial responses. Aneuploidy was reduced in the patients with dysplastic lesions. Oral pain and local ulcers developed in 52% of the patients (n = 26). Patients with a long history of OLK including hyperplasia and dysplastic lesions, as well as those with non-homogenous lesions, were more likely to develop pain and ulcer. During follow-up, the recurrence rate of hyperplasia and dysplastic lesions was 32% (n = 16) and the malignant transformation rate of dysplastic lesions was 4% (n = 2). Lesions on the buccal mucosa were associated with recurrence (*P* = 0.044; OR: 0.108, 95% CI: 0.013–0.915).

**Conclusion:**

Topical 5-ALA-mediated PDT is an effective treatment for OLK, particularly for homogenous leukoplakia, with few side effects. The buccal mucosa may be a protective factor that can reduce recurrence.

## Introduction

Owing to the increased risk of developing cancer in or near the lesion area, as well as in other parts of the oral cavity or head and neck region, oral leukoplakia (OLK) is listed as one of the oral potentially malignant disorders (OPMDs) [1]. A systemic review that included over 1,000 patients showed that the prevalence of OLK was between 1.49 and 4.27% [2]. Previous studies on leukoplakia indicated a wide range of malignant transformation rates (3–20%), and that this rate is increasing in the Chinese population [3, 4]. Although the main goal of OLK management is to prevent malignant transformation, the reduction or elimination of lesions is also desired because of their biological and clinical features [1, 5].

Various therapeutic approaches have been used in the management of OLK, such as systemic medication (alpha tocopherol, isotretinoin, lycopene, etc.), topical medication (bleomycin, tretinoin, isotretinoin, etc.), and photodynamic therapy (PDT) [6]. Although these approaches have been applied to OLK management, there is no clear evidence for an effective therapy preventing recurrence and malignant transformation [7].

Routes of administration of 5-aminolevulinic acid (5-ALA) include topical, oral, intravenous, intravesical, inhalational, and sublingual routes. Topical 5-ALA application is most widely used for mucocutaneous lesion PDT because it can be applied repeatedly without cumulative toxicity or serious adverse effects [8].

Topical PDT is a nonsurgical therapy using photosensitizers that selectively accumulate in the target tissue prior to light delivery. It involves three components: a light source, a photosensitizer, and tissue oxygen. The interaction between the light source and the photosensitizer is activated in the presence of oxygen, generating reactive oxygen species (ROS). Intracellular cytotoxic ROS then cause oxidative damage to precancerous and malignant cells [9, 10].

Topical PDT has been used for over 10 years for treating oral leukoplakia, with promising results [11]. In the study by Chen H.M. et al., 24 OLK patients were treated by topical PDT, with a result of 8 patients showing complete response and 16 patients showing partial response [12]. OLK recurred during the follow-up period in two cases. Kübler A et al. reported 12 OLK lesions treated by topical PDT with complete response in 5, partial response in 4, and no response in 3 patients while no recurrence was revealed [13]. However, data on the efficacy of topical PDT in OLK treatment for Chinese OLK patients are limited. There remain many barriers that lead to a significant proportion of patients with oral leukoplakia being treated with topical PDT. In this study, we reviewed the outcomes of OLK patients treated with topical PDT and evaluated the treatment effect, side effects, recurrence, and related risk factors. These findings provide novel insights into benchmarks and standards in the clinical application of topical PDT.

## Materials and methods

### Patients

Patients with OLK who received topical PDT in the Department of Oral Mucosal Diseases of the Affiliated Stomatological Hospital of Nanjing Medical University between January 2019 and October 2020 were recruited. Ethics committee approval was obtained prior to commencement of the study (PJ2019-115-001).

Fifty patients with untreated OLK were included in this study. The inclusion criteria were OLK patients clinically diagnosed and verified using histology (including hyperplasia and dysplasia), and aged 18–75 years [14, 15]. The exclusion criteria included a history of coagulopathy, pregnancy, porphyria, uncontrolled severe systemic illness such as heart disease, uncontrolled hypertension, severe liver and kidney injury, diabetes mellitus, or malignancy, and a history of allergy to light, porphyrins, or anesthetics [16]. All patients received guidelines limiting smoking and alcohol consumption, and oral hygiene instructions [15].

### Treatment procedures

The number of irradiated spots was determined according to the area of the lesion. An approximately 1 cm^2^ cotton piece was made with a sterile cotton swab for each spot. This was dipped into prepared 20% 5-ALA gel (Fudan Zhangjiang Bio-Pharmaceutical Co., Ltd., Shanghai, China) and used to cover the surface of the lesion; lesions and the surrounding circumference (0.3–0.5 cm) were completely covered. Cut rice paper (folded into three to four layers) and plastic wrap were successively used to cover the surface of the cotton piece, and then fixed with sterile gauze under pressure for another 2 h. Local infiltration anesthesia was administered with lidocaine. The illumination parameters used were a He-Ne laser (Leiyi Laser Technology Co., Ltd., Tianjin, China) of 635 nm and 150–300 mW/cm^2^, irradiation time of 300 s, and irradiation in a dark environment. The treatment course was three to four cycles with an interval of 7–14 days between each, depending on the healing of the lesion [16].

### Follow-up

Treatment response was typically recorded four weeks after the last treatment and was followed up every 3 months for the first year and every 6 months thereafter for up to 2 years. The treatment area was measured using a periodontal probe and was recorded using a digital camera. An experienced specialist in oral mucosal diseases performed the clinical examinations that included treatment response, side effects, clinical recurrence, and malignant transformation. Follow-up records were obtained using a chronic disease management service platform for OPMDs.

Treatment response was classified as complete response (CR, lesion disappearance), partial response (PR, lesion reduction of more than 20%), and no response (NR, lesion reduction of less than 20% or worsening) [16]. DNA ploidy analysis of exfoliated cells was performed to evaluate the efficacy of topical PDT. A special brush was used to rotate the biopsy site with a moderate force 10–15 times in the same direction; the brush was then placed in a preservation vial. The collected cells were then subjected to DNA ploidy analysis.

Side effects were assessed by telephone follow-up 1 week after topical PDT, and clinical recurrence was assessed at each follow-up visit except for the first visit. If the lesion showed the possibility of malignant transformation, a brush biopsy was done, and biopsy was performed if necessary. Patients completed follow-up and received treatment immediately if their lesions were converted to oral squamous cell carcinoma (OSCC) at any time during the study [17].

### DNA ploidy analysis

DNA ploidy was assessed using an automated DNA image cytometer (Landing Medical HingTech co., LTD, Wuhan, China). The negative diagnose suggested DNA euploid, whereas positive ones indicated that the DNA content of cells > 5c, or the hyperplasia cells from 2 to 4c accounting for over 10% of the total number of tested cells or showing aneuploidy peak [18, 19].

### Statistical analyses

SPSS 20 statistical software was used to analyze the data. Data were presented as number (percentage) or mean ± SD. Differences between groups were analyzed with Student’s t test or χ2 test. Logistic regression analysis was applied to evaluate the odds ratio (OR) and the association between variables, and the asymptotic 95% confidence interval (CI) of the OR was calculated. *P* < 0.05 was considered statistically significant.

## Results

### Demographic information

A total of 50 patients with OLK who met the inclusion criteria and received topical PDT (26 males and 24 females, mean age 55.5 ± 12.7 years) were included. Twenty-seven patients (54%, 27/50) were smokers and 32 patients (64%, 32/50) were drinkers. Of the 50 lesions, 40% (20/50) were observed on the floor of the mouth, followed by the buccal mucosa (28%, 14/50), margin surface of the tongue (10%, 5/50), palate (8%, 4/50), gingiva (6%, 3/50), dorsal surface of the tongue (4%, 2/50), and lip (4%, 2/50). Thirty-four lesions were observed to be homogenous and 16 were non-homogenous; of these lesions, 34% (17/50) were non-dysplastic (hyperplasia), 40% (20/50) were mildly dysplastic, 26% (13/50) were moderately to severely dysplastic. Detailed demographics of the patients are listed in Table 1.

**Table 1:** Demographics of patients who received topical photodynamic therapy

Variable	n (%) or mean ± SD
**Overall (n = 50)**	
**Sex**	
Female	24 (48.0%)
Male	26 (52.0%)
**Age (year)**	55.5 ± 12.7
**Smoking history**	
Yes	27 (54.0%)
No	23 (46.0%)
**Drinking history**	
Yes	32 (64.0%)
No	18 (36.0%)
**Lesion location**	
floor of the mouth	20 (40.0%)
buccal mucosa	14 (28.0%)
margin surface of the tongue	5 (10.0%)
palate	4 (8.0%)
gingiva	3 (6.0%)
dorsal surface of the tongue	2 (4.0%)
lip	2 (4.0%)
**Lesion classification**	
Non-homogenous	16 (32.0%)
Homogenous	34 (68.0%)
**Dysplastic pathology**	
Non-dysplastic	17 (34.0%)
Mildly dysplastic	20 (40.0%)
Moderately to severely dysplastic	13 (26.0%)
**Follow-up (month)**	20.3 ± 6.9

### Treatment response

**Fig. 1:**
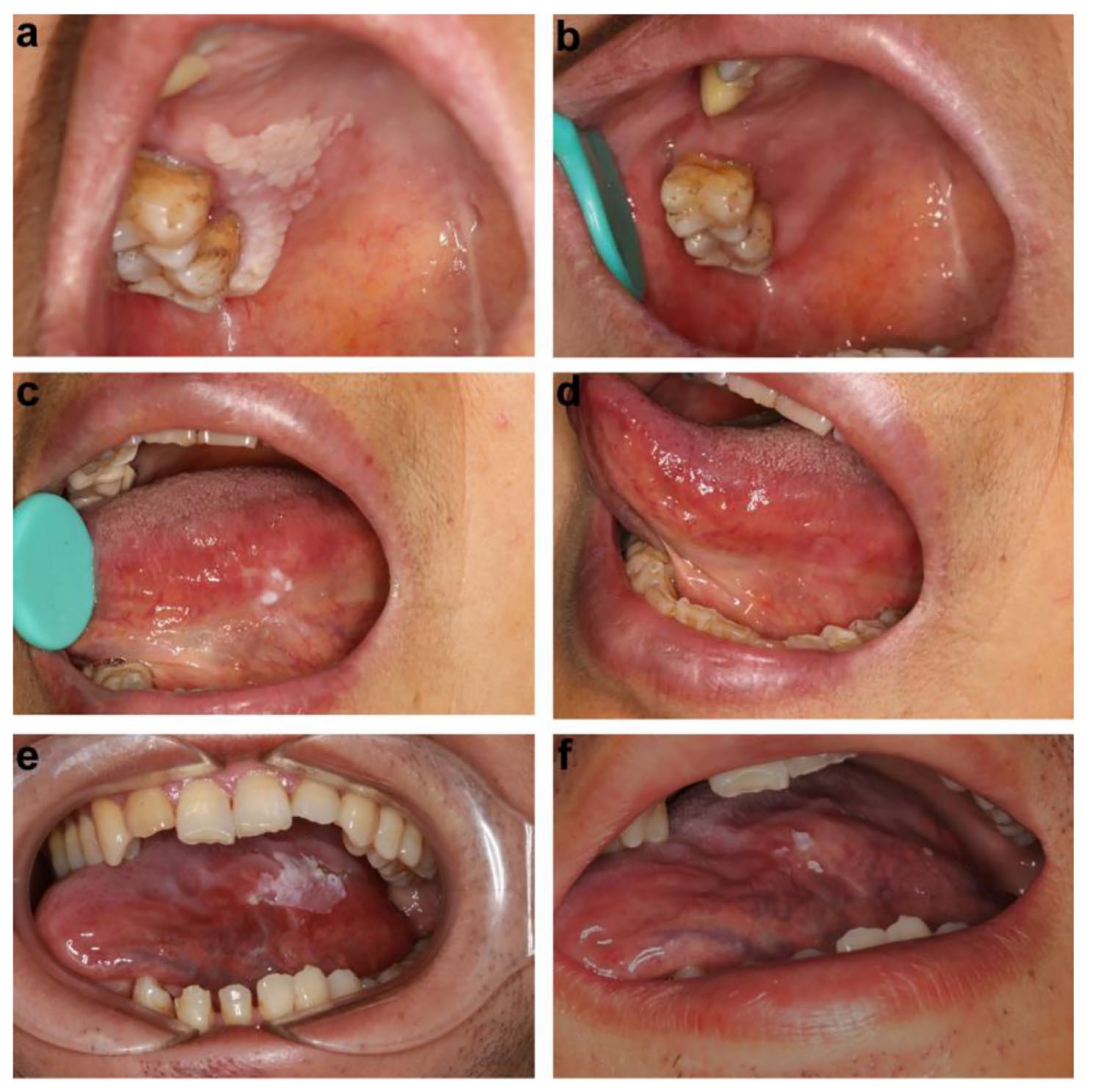
Representative pictures showing the different clinical outcomes. The oral leukoplakia lesion indicated a complete response (**a-d**) or a partial response (**e, f**) to photodynamic therapy

**Fig. 2:**
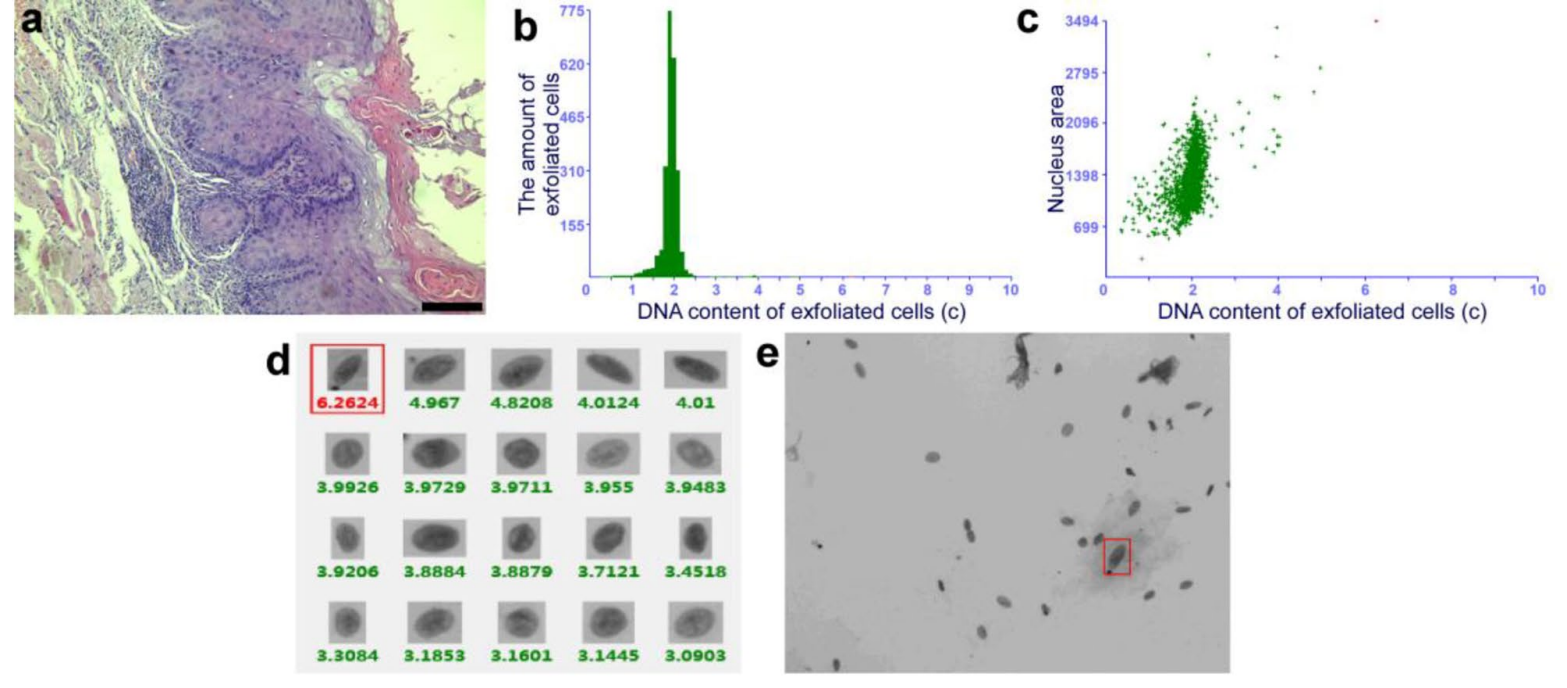
Histopathological examination and DNA ploidy analysis in the patient with dysplastic lesion before treatment. (**a**) The pathological changes of OLK before topical PDT were analyzed by hematoxylin-eosin (HE) staining (×100). Scale bar: 50 μm. (**b-e**) Brush biopsy with DNA-image cytometry, exfoliated cells taken from the margin surface of the tongue of a 56-year-old female subject

**Fig. 3:**
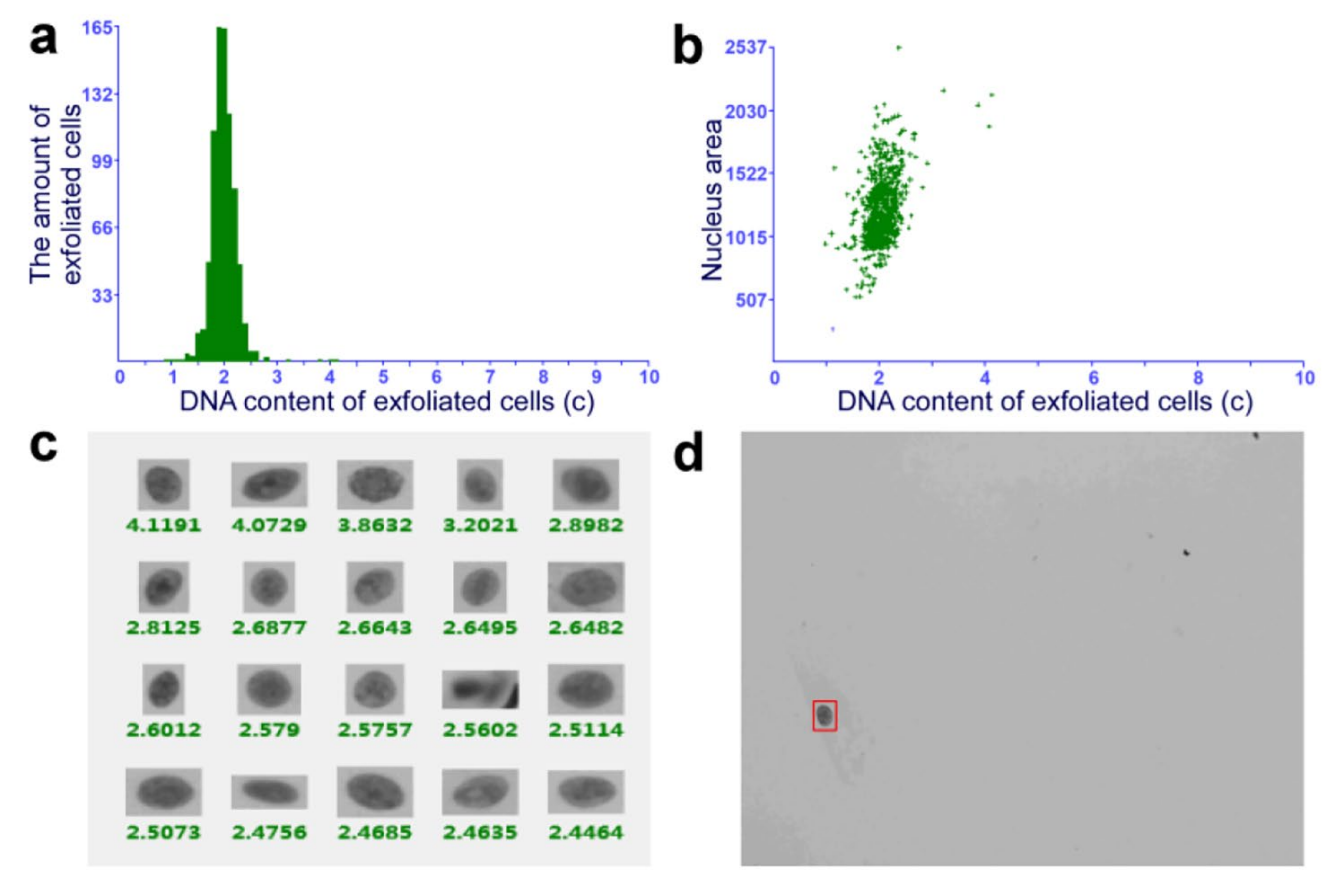
DNA ploidy analysis in the patient with moderate to severe dysplastic lesion after treatment. (**a-d**) Brush biopsy with DNA-image cytometry, exfoliated cells taken from the margin surface of the tongue of that 56-year-old female subject

**Fig. 4:**
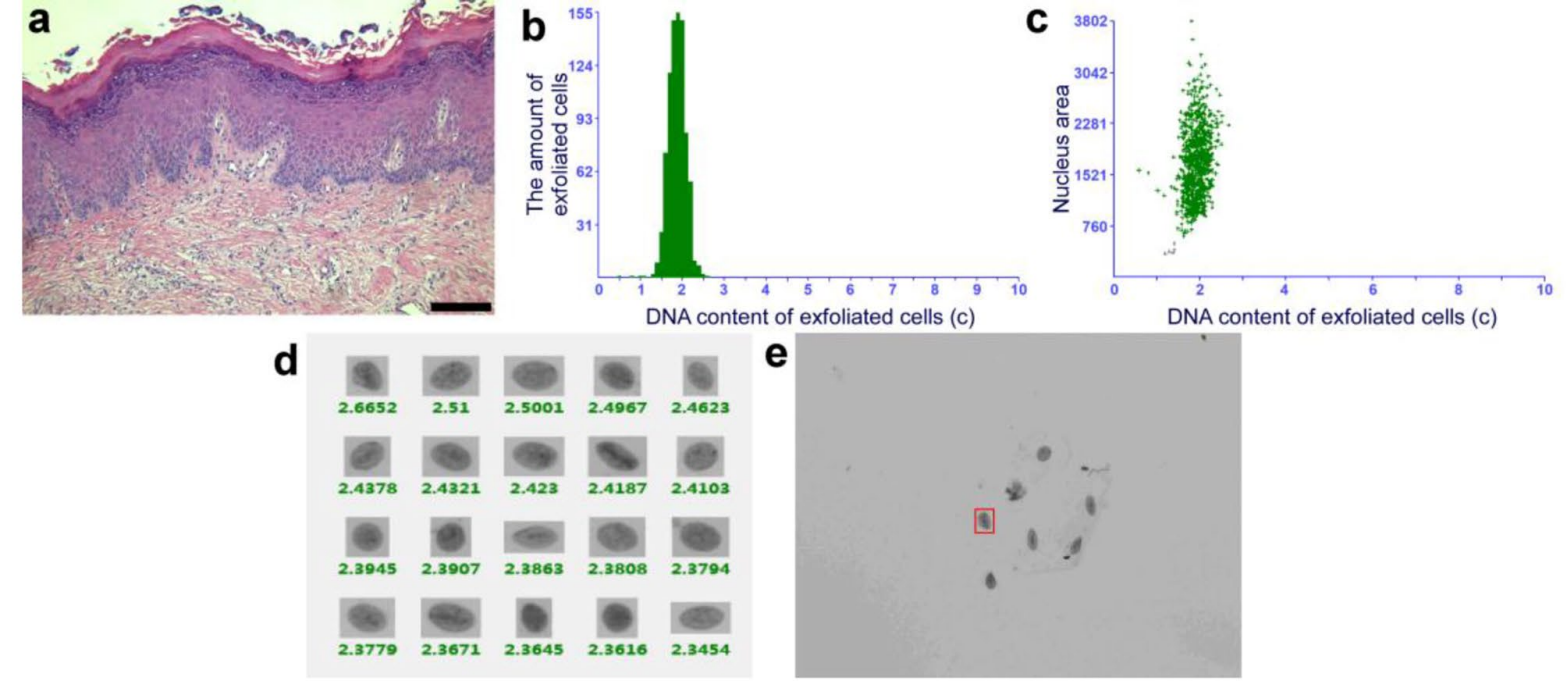
Histopathological examination and DNA ploidy analysis in the patient with non-dysplastic lesion before treatment. (**a**) The pathological changes of OLK before topical PDT were analyzed by HE staining (×100). Scale bar: 50 μm. (**b-e**) Brush biopsy with DNA-image cytometry, exfoliated cells taken from the palate of a 53-year-old female subject

**Fig. 5:**
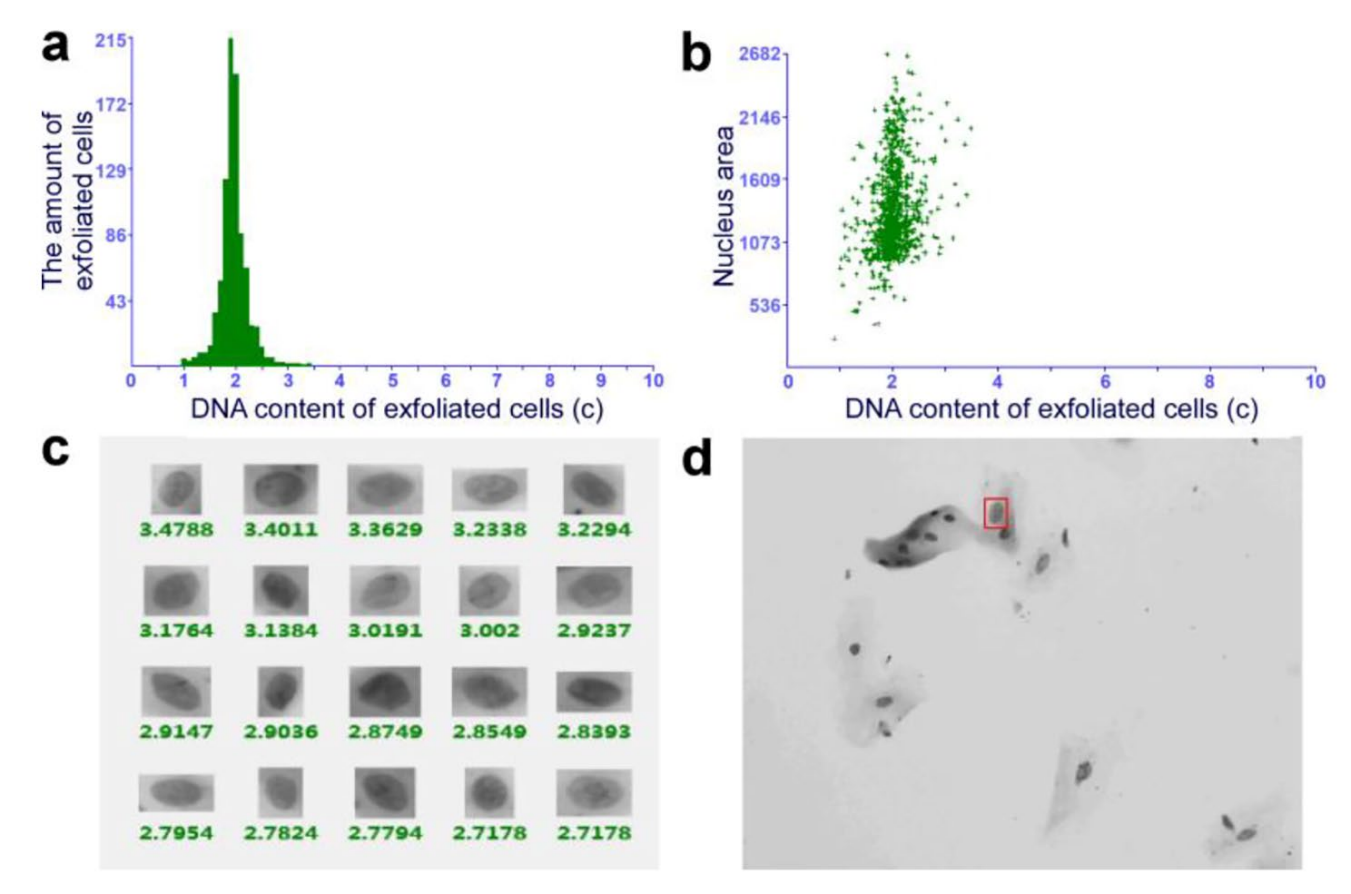
DNA ploidy analysis in the patient with non-dysplastic lesion after treatment. (**a-d**) Brush biopsy with DNA-image cytometry, exfoliated cells taken from the palate of that 53-year-old female subject

After topical PDT, 68% (34/50) of patients with OLK achieved a positive response, while 32% (16/50) did not show any change. Among the 34 responders, six and 28 patients achieved CR and PR, respectively (Fig. 1). When the oral tissue cytology of patients before and after treatment was examined, DNA ploidy analysis showed a reduction in aneuploidy after topical PDT in the patients with dysplastic lesions (Figs. 2 and 3). There was no change in aneuploidy after topical PDT in the patients with non-dysplastic lesions (Figs. 4 and 5). These results indicate that topical PDT has a positive effect on OLK patients.

The response rate to topical PDT significantly differed between homogeneous and non-homogeneous leukoplakia (*P* = 0.041, Table 2), suggesting a better clinical benefit in homogenous leukoplakia than in nonhomogeneous leukoplakia. In contrast, the efficacy of topical PDT was comparable among patients of different sexes, smoking history, drinking history or dysplastic pathology (*P* > 0.05, Table 2).

**Table 2:** Comparison of treatment responses in different patient groups

Characteristics	Treatment responses			
	CR	PR	NR	*P* value
**Total, n (%)**	6 (12.0%)	28 (56.0%)	16 (32.0%)	
**Sex**				
Female	4 (16.7%)	14 (58.3%)	6 (25.0%)	0.452
Male	2 (7.7%)	14 (53.8%)	10 (38.5%)	
**Smoking history**				
Yes	2 (7.4%)	15 (55.6%)	10 (37.0%)	0.473
No	4 (17.4%)	13 (56.5%)	6 (26.1%)	
**Drinking history**				
Yes	2 (6.25%)	20 (62.5%)	10 (31.25%)	0.209
No	4 (22.2%)	8 (44.4%)	6 (33.3%)	
**Lesion classification**				
Non-homogenous	1 (6.25%)	6 (37.5%)	9 (56.25%)	0.041*
Homogenous	5 (14.7%)	22 (64.7%)	7 (20.6%)	
**Dysplastic pathology**				
Non-dysplastic	2 (11.8%)	10 (58.8%)	5 (29.4%)	0.636
Mildly dysplastic	1 (5.0%)	12 (60.0%)	7 (35.0%)	
Moderately to severely dysplastic	3 (23.08%)	6 (46.15%)	4 (30.77%)	

### Side effects

Oral pain and local ulcers were observed in 52% of the patients (26/50) but were almost resolved within 2 weeks. These clinical side effects were examined and related to OLK history and classification, as patients with a long history of OLK as well as those with non-homogenous lesions were more likely to develop pain and ulcers (*P* < 0.05, Table 3). However, the side effect of topical PDT was comparable among patients of different dysplastic pathology (*P* > 0.05, Table 3).

**Table 3:** Comparison of side effects in different patient groups

Characteristics		Side effects	
	None or rough lesion texture	Oral pain or local ulcer	*P* value
**Total**, ***n*** **(%)**	24 (48.0%)	26 (52.0%)	
**OLK history (month)**	6.2 ± 5.0	17.1 ± 9.3	0.017*
**Lesion classification**			
Non-homogenous	4 (25.0%)	12 (75.0%)	0.009*
Homogenous	22 (64.7%)	12 (35.3%)	
**Dysplastic pathology**			
Non-dysplastic	9 (52.9%)	8 (47.1)	0.715
Mildly dysplastic	10 (50.0%)	10 (50.0%)	
Moderately to severely dysplastic	5 (38.5%)	8 (61.5%)	

### Clinical recurrence

**Fig. 6:**
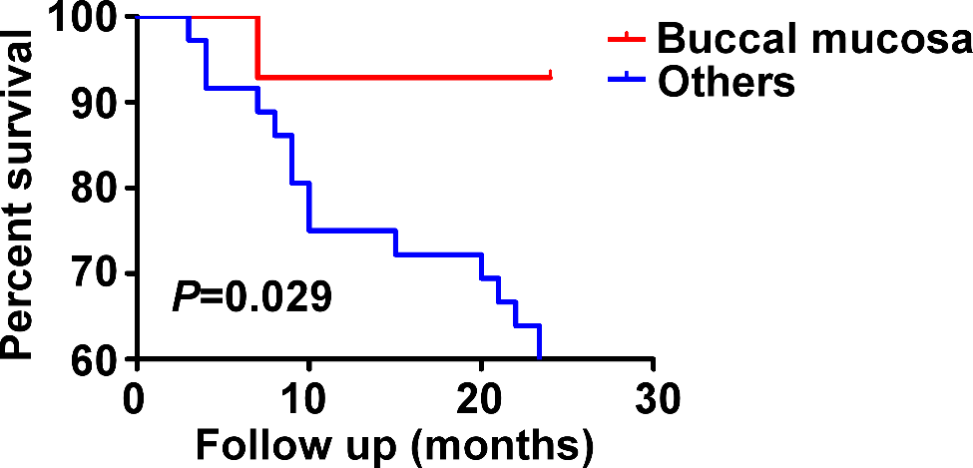
Kaplan–Meier curves for time to recurrence indicated the significance of the lesion location (*P* = 0.029)

During the 2-year follow-up period, 32% (16/50) of patients with OLK relapsed. Recurrence was observed in 16.7% (1/6) of the CR lesions, 46.4% (13/28) of the PR lesions, and 12.5% (2/16) of the NR lesions. The characteristics of the recurrence groups are presented in Table 4. Univariate analysis showed that sex, smoking and drinking history, and epithelial dysplasia were not statistically significant between those with recurrence and those without (*P* > 0.05, Table 4). Univariate analysis of time to recurrence and Kaplan–Meier curves (Fig. 6) indicated that buccal mucosa was a protective factor against recurrence (*P* = 0.044; OR: 0.108; 95% CI: 0.013–0.915; Table 4).

**Table 4:** Univariable analysis of risk factors for oral leukoplakia recurrence after topical photodynamic therapy

Characteristics	Non-recurrence	Recurrence	Univariable analysis
**Total,** ***n*** **(%)**	34 (68.0%)	16 (32.0%)	OR (95% CI)
**Gender**			*P* = 0.846
Female	16 (66.7%)	8 (33.3%)	1.0 (ref)
Male	18 (69.2%)	8 (30.8%)	1.125 (0.343–3.695)
**Smoking history**			*P* = 0.827
Yes	18 (66.7%)	9 (33.3%)	1.0 (ref)
No	16 (69.6%)	7 (30.4%)	1.143 (0.346–3.777)
**Drinking history**			*P* = 0.632
Yes	21 (65.6%)	11 (34.4%)	1.0 (ref)
No	13 (72.2%)	5 (27.8%)	1.362 (0.385–4.817)
**Lesion location**			*P* =0.044*
Other location	21 (58.3%)	15 (41.7%)	1.0 (ref)
Buccal mucosa	13 (92.9%)	1 (7.1%)	0.108 (0.013–0.915)
**Dysplastic pathology**			*P* = 0.408
Non-dysplastic	13 (76.5%)	4 (23.5%)	1.0 (ref)
Mildly dysplastic	14 (70.0%)	6 (30.0%)	1.393 (0.319–6.078)
Moderately to severely dysplastic	7 (53.8%)	6 (46.2%)	2.786 (0.583–13.305)

### Malignant transformation

During the follow-up, the cumulative incidence of malignant transformation was 4% (2/50). The clinicopathological parameters of each patient with malignant transformation are presented in Table 5.

**Table 5:** Clinicopathological parameters of patients with malignant transformation

	Gender	Age (year)	Smoking history	Drinking history	Lesion location	Dysplastic pathology	Malignant transformation time (month)
1	Female	65	No	No	floor of the mouth	Severely dysplastic	3
2	Male	57	Yes	Yes	floor of the mouth	Moderately dysplastic	18

## Discussion

Topical PDT has been proven effective for the treatment of OLK. A systematic review showed that 7.7–90.9% of lesions in patients treated with topical PDT had a complete response, whereas 0–66.7% of lesions showed a partial response. Overall, the complete and partial response rates were 32.9% and 43.2%, respectively, with a combined response rate of 76.1% [20]. In this study, the complete and partial response rates were 12% and 56%, respectively, indicating a high overall response rate.

Despite this, the findings of patients who do not respond to topical PDT and those who relapse prove the limitations of this therapy. Li et al. found that 0–59% of patients did not respond to topical PDT [20]. The non-response rate in this study was 32%, which is comparable to the results of previous studies. Studies have shown that non-response may be due to a variety of reasons. In a study by Wong et al., the absence of response to topical PDT was attributed to a low-energy light source of 2–4 J/cm^2^, as the common energy used is approximately 100 J/cm^2^ [21]. In addition, limited penetration depth of the light source, large lesion areas, and incomplete treatment can also contribute [22].

Chen et al. found that after administrating topical PDT, patients experiencing OLK lesions with mild or moderate dysplasia exhibited a better response than those without dysplasia due to wide intercellular spaces of the dysplastic epithelium. This reduces the keratotic epithelial surface and makes the epithelium in dysplastic lesions thinner and more permeable [20]. However, this study showed no significant difference among patients with different dysplastic pathology, which could be due to the small sample size. Moreover, this study found that topical PDT performed better in treating homogeneous lesions than non-homogeneous lesions, which was consistent with most studies [23]. Non-homogeneous leukoplakia has a higher malignant transformation rate than homogeneous leukoplakia because of epithelial dysplasia [24]. Non-homogeneous leukoplakia is more prone to moderate and severe dysplasia than homogeneous leukoplakia, leading to lower treatment efficacy.

OSCC is a continuous condition that includes atypical hyperplasia, carcinoma in situ, and invasive carcinoma. Brush biopsy and DNA aneuploidy detection have high sensitivity and specificity for detecting malignant transformation of the oral epithelium, using a painless and non-invasive process that is easy to perform. Compared with the standard “scalpel biopsy” diagnostic method, the biopsy analysis of DNA has higher compliance. DNA aneuploidy is an internationally recognized marker for tumor cell transformation and is the cytological equivalent of chromosomal aneuploidy [18, 25]. Through DNA ploidy analysis, the condition of OPMD can be determined along with whether malignant transformation has occurred [26]. The results of this study showed a reduction in aneuploidy after topical PDT in the patients with dysplastic lesions, suggesting a downward trend in the association between topical PDT and the evolution of oral precancerous or malignant lesions.

The long observation period makes it possible to monitor and identify the recurrence in treated sites. The reported recurrence rate of OLK to topical PDT varied from 0 to 41% over a follow-up period of 1–30 months [27–29]. The 32% recurrence rate in this study was comparable to that reported in other studies. In addition, buccal mucosal lesions showed a lower risk of recurrence than other lesions. Pietruska et al. reasoned that topical PDT was more effective for lesions on the cheek and lip than for lesions on the tongue and gums [29]. We speculate that this may have resulted from less friction and pressure during chewing [30]. Current strategies for OLK surveillance include a “wait-and-watch” method and histopathological identification by biopsy, vital staining, light-based techniques, cytology and molecular markers [31]. However, the visual interpretation of OLK evolution would be subjective, probably resulting in unnecessary invasive manipulation and/or delays in OSCC diagnosis [32]. DNA ploidy analysis could be an effective noninvasive method to objectively surveillance the photodynamic prognosis of high-risk OLK [33].

Topical 5-ALA application is more convenient than intravenous photosensitizers for patients. In addition, topical 5-ALA use can avoid hypotension caused by oral 5-ALA use in addition to avoiding systemic photosensitization caused by intravenous photosensitizers [34]. Most studies using lasers reported side effects or adverse reactions of the therapy. Patients usually suffered from burning, pain, tissue edema, and erythema in treated sites for a period of time, and some developed ulcers and loss of sensation [23]. The most common adverse effects of topical PDT observed in this study were pain and ulcers in the treated area and surrounding tissues. Almost all the patients tolerated these side effects. Pain or bleeding from the ulcer interferes with medication use; therefore, for patients who do not recover within two weeks, the treatment interval can be appropriately extended [15]. Photofrin and Foscan are two photosensitizers that are more effective than 5-ALA. They are usually used to treat oral cancers rather than OPMDs because they have higher penetration depth. These factors need to be taken into account before applying PDT [35].

The World Health Organization Working Group defines oral potentially malignant disorders (OPMDs) as all clinical features with a risk of oral cancer formation, which include oral leukoplakia, oral lichen planus, erythroleukoplakia and oral verrucous hyperplasia [36]. The primary goal of OLK is to prevent malignant transformation. The rate of malignant transformation in this study was 4%, which is comparable to the rate of OLK malignancy reported in other studies [37]. Owing to the short follow-up time and relatively small sample size, the effect of topical PDT on OLK malignant transformation could not be assessed in this study. However, it should be noted that both malignant changes occurred on the floor of the mouth, which is considered a high-risk site of OLK [38, 39]. In addition, moderately to severely dysplastic lesions were more likely to undergo malignant transformation than mildly dysplastic lesions and non-dysplastic lesions.

## Conclusion

In summary, topical 5-ALA-mediated PDT appears to be an effective treatment for OLK, especially for homogenous leukoplakia, with few side effects. The buccal mucosa may be a protective factor for reducing recurrence. The effect of topical PDT on malignant transformation should be further studied to improve its efficacy for OLK treatment. Randomized controlled trials with longer follow-up periods, standardized topical PDT parameters, and comparisons of the efficacy of topical PDT with various therapies are needed to obtain definitive conclusions.

## Data Availability

All data analysed in this study are included in this published article.

## Abbreviations

PDT: photodynamic therapy
OLK: oral leukoplakia
5-ALA: 5-aminolevulinic acid
OPMDs: oral potentially malignant disorders
ROS: reactive oxygen species
CR: complete response
PR: partial response
NR: no response
OSCC: oral squamous cell carcinoma
OR: odds ratio
CI: confidence interval
